# Editorial: Medico-legal aspects of clinical risk management and patient safety

**DOI:** 10.3389/fpubh.2022.970258

**Published:** 2022-09-05

**Authors:** Davide Ferorelli, Matteo Bolcato, Anna Aprile, Alessandro Dell'Erba

**Affiliations:** ^1^Section of Legal Medicine, Department of Interdisciplinary Medicine, University of Bari, Bari, Italy; ^2^Department of Neuroscience, University of Padua, Padua, Italy; ^3^Legal Medicine, University of Padua, Padua, Italy

**Keywords:** safety, clinical risk management, patient safety, legal medicine, litigation

From the 1990s to date, clinical risk management has become an increasingly important issue in healthcare systems (1–3). Since the publication of “To err is human” (4) in 2000, scientific evidence has shown the need for a new approach to handling adverse events in health systems. As a result, a new “no blame” culture has developed, and Clinical Risk Management has assumed a crucial role in healthcare systems because of the inherent repercussions on patient safety, optimization of clinical outcomes for patients, and reallocation of resources.

Over the years, healthcare safety has become a constituent element of the right to health for citizens in many nations. Consequently, clinical risk management has become even more essential, which has simultaneously generated an interesting debate on the numerous medico-legal aspects that have arisen and that will continue to present themselves over time.

Today more than ever, the recent development of the application areas of clinical risk management highlights very delicate issues such as medical professional liability, management of medico-legal disputes, informed consent, physician-assisted suicide, prevention of nosocomial infections, development of telemedicine, humanization of care, communication of errors, complaint management, and many other issues of great interest.

The foregoing has led to the need for 2-fold care and reflection: firstly, the need to create an integrated approach to clinical risk management in which the safety of care can only be achieved by guaranteeing the safety of patients, healthcare professionals, and hospitals.

Secondly, it cannot be denied that in some nations clinical risk management is moving further and further away from the original concept of a “no blame” culture and patient safety, resulting in medico-legal aspects assuming increasing importance. Such observations should stimulate the entire scientific community, as it did the authors of the articles published in this special issue, to endeavor to understand and interpret the phenomenon we are witnessing.

Ferorelli et al. analyzed the topic of adverse events suffered by patients during surgery, emphasizing the importance of using the surgical checklists as a simple tool to help reduce surgical adverse events. The authors found that adherence to surgical checklists is often lacking as a result of the cultural perception that checklists represent an extra administrative duty rather than an opportunity to promote patient safety. Their study assessed and demonstrated the efficacy of a free intervention, such as a short training course on risk management and safety checklists in order to improve checklist compliance.

De Donno et al. analyzed the ethical and medico-legal point of view of the suspension of care for patients with spasticity during the COVID-19 pandemic. Their findings highlighted how the COVID-19 pandemic has revolutionized the habits of entire communities, with profound negative effects on healthcare for the chronically ill. The study discussed the ethical dilemmas and unintended consequences of healthcare systems which have changed their priorities during the pandemic, directing almost all available resources toward the care of COVID-19 patients.

Serafimovska et al. proposed a study with the objective of evaluating the impact of cannabis extract obtained from cannabis flowers with maximum allowed residual level of aflatoxins and ochratoxin on human health and safety. The authors demonstrated that mycotoxins, especially aflatoxins, which are extremely toxic secondary metabolites, can reach critical levels in cannabis extracts obtained from dry cannabis flowers with the maximum quantity allowed of mycotoxins and that this can pose a great risk to consumers and their health, especially to those with compromised immune systems.

D'Errico et al. made proposals to improve the quality and safety of care through Clinical Risk Management in the context of medication errors in pediatrics that represent one of the most common causes of adverse events in pediatrics and are widely reported in the literature. The authors highlighted that although reports of literature are comfortable, additional research is needed to identify optimal strategies for developing a prevention-oriented risk culture based on the belief that errors, if detected and analyzed correctly, represent a precious opportunity for improvement.

La Russa et al. proposed a pilot project for hemodialysis facilities using the proactive risk assessment through Failure Mode and Effect Analysis (FMEA). The application of the FMEA process has shown that specific strategies for each failure mode must also be listed, as regards improvement activities from a clinical, organizational, and training point of view, in order to build a Risk Management Plan for hemodialysis facilities.

Tattoli et al. analyzed the role of “third-party” commission in the risk assessment and management for potential living kidney donors, highlighting that the selection and assessment of donor eligibility must follow appropriate standards. These standards should include a specific and informed consent process which makes the potential donors aware of the risks of their upcoming procedure, in addition to their prospects of life with only one kidney. The analysis showed that particular attention must be paid to the potential donor as the person most exposed to ethical risks.

Silvestre et al. carried out a comparative analysis on Italian legislation in the European context on the provision of advance treatment directives (DAT), hoping to generate uniformity in the discipline that regulates the expression of a patient's will as far as treatment is concerned. The objective was to avoid differences in both the rights and duties of the various roles involved in the healthcare path.

Bolcato et al. analyzed the guiding principles for surgical pathways intended as a tool for improving outcomes and patient safety. The authors reported that the literature agrees on the importance of combined systems of clinical risk management and lean management in surgical pathways. They also reported that the safety objectives are achievable by means of the adoption of structured organizational paths which include, among others, pre-hospitalization and the application of patient blood management.

## Author contributions

DF and AD wrote the first draft of the manuscript. AA and MB contributed to conception and design the editorial. All authors contributed to the article and approved the submitted version.

## Conflict of interest

The authors declare that the research was conducted in the absence of any commercial or financial relationships that could be construed as a potential conflict of interest.

## Publisher's note

All claims expressed in this article are solely those of the authors and do not necessarily represent those of their affiliated organizations, or those of the publisher, the editors and the reviewers. Any product that may be evaluated in this article, or claim that may be made by its manufacturer, is not guaranteed or endorsed by the publisher.
